# Ethanol Extract of *Lepidium apetalum* Seed Elicits Contractile Response and Attenuates Atrial Natriuretic Peptide Secretion in Beating Rabbit Atria

**DOI:** 10.1155/2013/404713

**Published:** 2013-10-29

**Authors:** Seung Ju Kim, Hye Yoom Kim, Yun Jung Lee, Hao Zhen Cui, Ji Yeon Jang, Dae Gill Kang, Ho Sub Lee

**Affiliations:** ^1^School of Oriental Medicine & College of Oriental Medicine, Wonkwang University, Iksan, Jeonbuk 570-749, Republic of Korea; ^2^Hanbang Body-Fluid Research Center, Wonkwang University, Iksan, Jeonbuk 570-749, Republic of Korea; ^3^The College of Chinese Traditional Medicine, Yanbian University, Yanji, Jilin 133002, China

## Abstract

The seeds of *Lepidium apetalum* Willdenow (called “Tinglizi” in China and “Jungryukza” in Korea) have been used to discharge phlegm and improve dropsy in Oriental medicine. The present study investigated the effects of ethanol extract of the seeds of *Lepidium apetalum* (ELA) on atrial dynamics and atrial natriuretic peptide (ANP) secretion in beating rabbit atria. ELA increased atrial stroke volume, pulse pressure, and cAMP efflux, concomitantly attenuating ANP secretion in a dose-dependent manner. ELA-induced increases in atrial stroke volume, pulse pressure, and cAMP levels and decrease in ANP secretion were not inhibited by pretreatment with staurosporine, a nonspecific protein kinase inhibitor, or diltiazem and verapamil, the L-type Ca^2+^ channel blockers, respectively. Helveticoside, a well-known digitalis-like cardiac glycosidic constituent of ELA, also increased atrial dynamics, including stroke volume and pulse pressure, without changing cAMP efflux and ANP secretion, and the effects of helveticoside were not inhibited by pretreatment with staurosporine, diltiazem, and verapamil. These results suggest that the ELA-induced positive inotropic activity in beating rabbit atria might, at least partly, be due to the digitalis-like activity of helveticoside rather than an increase in cAMP efflux.

## 1. Introduction

Cardiac glycosides are a diverse family of naturally derived compounds that bind to and inhibit Na^+^/K^+^-ATPase. Members of this family have been used for many years for the treatment of heart failure and atrial arrhythmia, and the mechanism of their positive inotropic effect is well characterized. There are many different well-described clinical trials of drugs for the treatment of chronic heart failure, including cardiac glycosides, sympathomimetics, phosphodiesterase (PDE) III inhibitors, diuretics, and angiotensin-converting enzyme inhibitors [1]. Stimulation of *β*-adrenergic receptor with a sympathomimetic agent induces positive inotropic effects, which are dependent on protein kinases (PKs) and L-type Ca^2+^ channels [2, 3]. The increase in cyclic adenosine monophosphate (cAMP) levels induced by PDE III inhibitors also accentuates cardiac contractility via activation of protein kinases and L-type Ca^2+^ channels [4]. Treatment of heart failure patients with cardiac glycosides like digitalis, which augment pump function by increasing the contractility of cardiac myocytes, is known to improve hemodynamics and exercise tolerance [5, 6]. In the regulation of cardiac contractility, Ca^2+^ plays a pivotal role and has been implicated in the functional mechanism of various agents involved in the modulation of cardiac action [7, 8]. In addition, several signal transduction factors like cAMP, inositol triphosphate (IP_3_), diacylglycerol (DAG), PK, and adenylyl cyclase (AC) influence the generation of cardiac contractile forces and regulate intracellular Ca^2+^ concentrations [9].

Atrial natriuretic peptide (ANP) is synthesized and stored in atrial cardiomyocytes and secreted into the bloodstream by atrial stimulation [10]. The secretion of ANP from cardiomyocytes under mechanical stimulation has been known to regulate body fluid levels through relaxation of vascular smooth muscle and inhibition of water and renal electrolyte reabsorption [11].

The seeds of* Lepidium apetalum* Willdenow (Cruciferae, called “Tinglizi” in China and “Jungryukza” in Korea) have been used to discharge phlegm and improve dropsy in Oriental medicine. From the seeds of *Lepidium apetalum*, compounds such as helveticoside, linoleic acid, and olein have been isolated [12]. Recently, it was reported that an extract of the seeds of *Lepidium apetalum* inhibits skin pigmentation mediated by IL-6-driven signaling. However, to the best of our knowledge, the inotropic effect of *Lepidium apetalum *in perfused beating atria has not been defined. Therefore, we performed this study to elucidate the mechanism of ELA-induced positive inotropic activity in perfused beating rabbit atria.

## 2. Materials and Methods

### 2.1. Plant Materials and Extraction

The seeds of *Lepidium apetalum* Willdenow were commercially available and purchased from the herbal market in Iksan, Jeonbuk Province, and authenticated by professor Tae-Oh Kwon, College of Life Sciences and Natural Resources, Wonkwang University. A herbarium voucher specimen (HBI-048) was deposited in the herbarium of the Professional Graduate School of Oriental Medicine, Wonkwang University, Iksan, Jeonbuk, South Korea. The dried seeds of *Lepidium apetalum *(600 g) were subjected to extraction procedures with 1 L of 95% ethanol thrice, with each extraction being performed for 24 h. The ethanol extract was filtered through a Whatman No. 3 filter paper, concentrated using a rotary evaporator (ELA, 1.5 g), and then used in experiments.

### 2.2. Preparation of Perfused Beating Rabbit Atria and Determination of Atrial Stroke Volume and Pulse Pressure

New Zealand white male rabbits weighing 2 kg were used as the source of rabbit atria. Each rabbit was anesthetized by injecting ketamine-HCl, and its chest was opened. An isolated perfused atrial preparation was prepared by a slightly modified version of Cho's method [13], allowing atrial pacing and measurements of changes in atrial volume during contraction (stroke volume) and cAMP efflux. Briefly, the hearts were rapidly removed and placed in oxygenated warm saline. The left atrium was then dissected. A calibrated transparent atrial cannula containing 2 small catheters was inserted into the left atrium through the atrioventricular orifice. The cannula was secured by ligatures around the atrioventricular sulcus. The outer tip of one of the 2 catheters located in the atrium was used for perfusion, and the other catheter was used to record pressure changes in the atrium. The cannulated atrium was then transferred to an organ chamber containing 3 mL of buffer at 34°C. The pericardial space of the organ chamber was opened to air so as not to restrict atrial dynamics. The atrium was immediately perfused with N-2-hydroxyethylpiperazine-N′-2-ethanesulfonic acid (HEPES) buffer solution by means of a peristaltic pump (1 mL/min). The buffer was prepared using the following constituents: 118 mM NaCl, 4.7 mM KCl, 2.5 mM CaCl_2_, 1.2 mM MgCl_2_, 25 mM NaHCO_3_, 10.0 mM glucose, and 10.0 mM HEPES (adjusted to pH 7.4 with 1 M NaOH) and 0.1% bovin serum albumin (BSA). Soon after setup of the perfused atrium, transmural electrical field stimulation at 1.3 Hz (duration, 0.3–0.5 ms; voltage, twice the threshold intensity, 20–30 V; distention, 6.1 cm H_2_O) was started with a luminal electrode. The organ chamber was fixed so as to allow axial rotation to change the height of the atrial cannula and intra-atrial pressure. The perfusate was prewarmed to 34°C by passage through silicone tubing in a mixed gas chamber. The buffer in the organ chamber was oxygenated.

### 2.3. Measurement of ANP Levels in Perfusates

The levels of immunoreactive ANP in the perfusate were measured by radioimmunoassay, as previously described [13]. The radioimmunoassay was performed in tris (hydroxymethyl) aminomethane (Tris)-acetate buffer (0.1 mM EDTA, 0.005% soybean trypsin inhibitor, 0.02% sodium azide, 0.0004% phenylmethylsulfonyl fluoride, and 1% BSA, at pH 7.4). The sample volume used for radioimmunoassay was 50 *μ*L, and the total assay volume was 300 *μ*L. Standard or perfusate samples were incubated with 100 *μ*L of anti-ANP antibody and 100 *μ*L of ^125^I-labeled ANP for 24 h at 4°C. The separation of free tracer from antibody-bound tracer was achieved by adding 1.0 mL of dextran-charcoal suspension (charcoal, 6.0 g; Dextran T-70, 0.625 g; phenylmercuric acetate, 34 mg; and neomycin, 2 g in 1 L of Tris-acetate buffer, 0.1 M, pH 7.4). Radioimmunoassay for ANP was performed on the day of the experiments, and all samples in an experiment were analyzed in a single assay. The secreted amount of ANP was expressed as nanograms of ANP per minute per gram of atrial wet weight.

### 2.4. Preparation of Samples for cAMP Assay

To prepare the perfusates for cAMP assay, 100 *μ*L of the perfusate was treated with trichloroacetic acid (100 *μ*L) to a final concentration of 6% for 15 min at room temperature and centrifuged at 4°C. The supernatant (100 *μ*L) was transferred to a polypropylene tube, extracted 3 times with water-saturated ether (300 *μ*L), and dried using a speedVac concentrator (Savant, Farmingdale, NY, USA). The dried samples were resuspended in 50 mM sodium acetate buffer (pH 4.85).

### 2.5. Measurement of cAMP Levels in Perfusates

Production of cAMP was measured in an equilibrated radioimmunoassay, as described previously [14]. Briefly, standards or samples were made up to a final volume of 100 *μ*L in 50 mM sodium acetate buffer (pH 4.8) containing theophylline (8 mM). Then, 100 *μ*L of diluted cAMP antiserum and iodinated 2′-O-monosuccinyl-adenosine 3′,5′-cyclic monophosphate tyrosyl methyl ester (^125^I-ScAMP-TME, 10,000 counts/min [cpm] per 100 *μ*L) were added, and the mixture was incubated for 24 h at 4°C. For the acetylation reaction, 5 *μ*L of a mixture of acetic anhydride and triethylamine (1 : 2 dilution) was added to the assay tube before adding antiserum and tracer as well. The bound form was separated from the free form by charcoal suspension. ^125^I-ScAMP-TME was prepared as described previously [15]. Briefly, 2 *μ*g of ScAMP-TME was introduced into a vial containing 100 mM phosphate buffer (pH 7.4), and 1 mCi of ^125^I-Na was added. Chloramine-T (0.4 mg/mL) was added to the reaction vial (total reaction volume = 50 *μ*L) and mixed gently, and 1 min later, the reaction was terminated with sodium metabisulfite (0.2 mg/mL) and NaI^125^ (5 mM). The reaction mixture was immediately applied to a Sephadex G-10 column (1 × 20 cm) previously washed with 10 mM phosphate buffer (pH 7.4). ^125^I-ScAMP-TME was eluted with 10 mM phosphate buffer containing 150 mM NaCl (pH 7.4) and stored at −20°C until further use. Immediately before it was used, ^125^I-ScAMP-TME was repurified by high-performance liquid chromatography (HPLC) on a reversed-phase *μ*Bondapak column (Waters Associates, Milford, MA, USA) with a linear gradient (0–60% acetonitrile in 0.1% trifluoroacetic acid). Radioimmunoassay for cAMP was performed on the day of the experiments, and all samples from one experiment were analyzed in a single assay. Nonspecific binding was <2.0%. The 50% intercept was at 16.50 ± 0.79 fmol/tube (*n* = 10). The amount of cAMP was expressed as picomole per minute per gram of atrial tissue.

### 2.6. Measurement of K^+^ Concentration in Perfusates

Before and after the perfusion of beating rabbit atria with HEPES buffer, the K^+^ concentration in the perfusates was measured by using an electrolyte analyzer (NOVA 5, Biochemical, Waltmam, MA, USA) and expressed as mmol/L.

### 2.7. Reagents

HEPES, sodium chloride, potassium chloride, calcium chloride, magnesium chloride, sodium bicarbonate, glucose, BSA, sodium acetate, aprotinin, glycine, lysozyme, theophylline, sodium azide, potassium phosphate monobasic, potassium phosphate dibasic, charcoal, diltiazem, verapamil, ouabain, and helveticoside were purchased from Sigma Chemical Co. (St. Louis, MO, USA). The following reference materials were obtained from the sources specified: anti-cAMP (Merck Bioscience Calbiochem, USA), anti-ANP (Homemade, Korea), staurosporine (Biomol Research Laboratories Inc, USA), and ^125^I-Na (Amersham Biosciences, Sweden). Stock solutions of diltiazem, verapamil, staurosporine, and helveticoside were prepared in DMSO. Control experiments demonstrated that the highest DMSO level (0.2%) had no effect on beating rabbit atria.

### 2.8. Statistical Analysis

The results are shown as means ± SE. Data was analyzed by repeated measures ANOVA followed by Bonferroni's multiple-comparison test. Student's *t*-test for unpaired data was also applied. Statistical significance was defined as *P* < 0.05.

## 3. Results

### 3.1. Effect of ELA on the Atrial Dynamics, cAMP Efflux, and ANP Secretion

**Figure 1:**
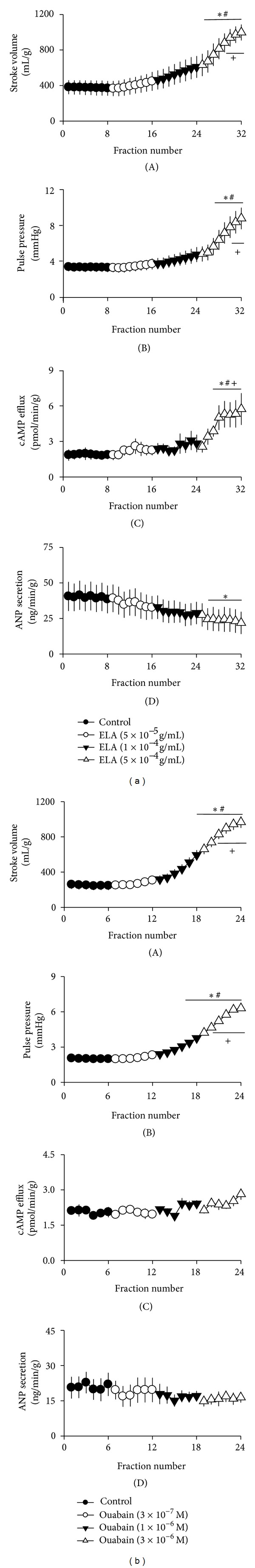
Dose-response curves of ELA (a) and ouabain (b) for stroke volume (A), pulse pressure (B), cAMP efflux (C), and ANP secretion (D) in beating rabbit atria. Values shown are mean ± SE (*n* = 4); ^+^ *P* < 0.05 versus control; **P < 0.01 versus ELA (5 × 10^−5^ g/mL) or ouabain (3 × 10^−7^ M); ^###^ *P* < 0.001 versus ELA (1 × 10^−4^ g/mL) or ouabain (1 × 10^−6^ M) (compared with values for the last 3 fractions of control).

In beating rabbit atria, treatment with ELA increased stroke volume and pulse pressure in a dose-dependent manner (Figures 1(a)(A) and 1(a)(B)). Treatment with ELA also increased cAMP efflux in beating rabbit atria (Figure 1(a)(C)). On the other hand, treatment with ELA markedly decreased ANP secretion in beating rabbit atria (Figure 1(a)(D)). Ouabain, which was used as a positive control, significantly increased stroke volume (Figure 1(b)(A)) and pulse pressure (Figure 1(b)(B)), with no change in cAMP efflux (Figure 1(b)(C)) and ANP secretion (Figure 1(b)(D)).

### 3.2. Effect of Staurosporine on ELA-Induced Changes

**Figure 2:**
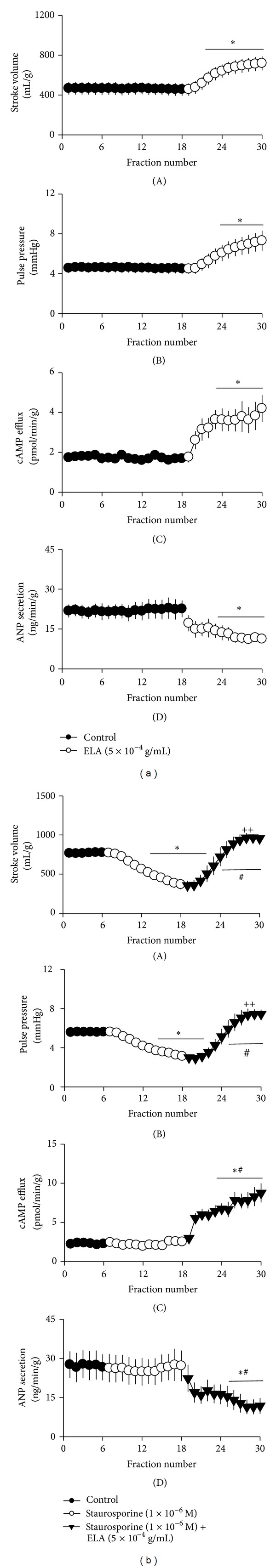
Effects of ELA (a) and staurosporine (b) on ELA-induced changes in stroke volume (A), pulse pressure (B), cAMP efflux (C), and ANP secretion (D) in beating rabbit atria (1.3 Hz). Values shown are mean ± SE (*n* = 4); ***P < 0.001 versus control; ^###^ *P* < 0.001 versus staurosporine (compared with values for the last 3 fractions of control or staurosporine).

To define the role of protein kinases in the ELA-induced positive inotropic effect, the effects of staurosporine, a nonspecific PK inhibitor, on beating rabbit atria were tested. Treatment with ELA (5 × 10^−4^ g/mL) induced an increase in stroke volume, pulse pressure, and cAMP efflux and a decrease in ANP secretion in beating rabbit atria (Figures 2(a)(A), 2(a)(B), 2(a)(C), and 2(a)(D)). Treatment of beating atria with staurosporine (1 × 10^−6^ M) significantly decreased stroke volume and pulse pressure, in comparison with the corresponding levels in controls (Figures 2(b)(A) and 2(b)(B)). However, subsequent treatment with ELA (5 × 10^−4^ g/mL) reverted the changes in atrial stroke volume and pulse pressure and increased the values to levels much higher than basal levels (Figures 2(b)(A) and 2(b)(B)). Staurosporine did not affect cAMP efflux in beating atria. However, ELA substantially increased cAMP efflux in the staurosporine-pretreated atrium (Figure 2(b)(C)).

In addition, staurosporine had no effect on ANP secretion in beating atria. However, ELA markedly decreased ANP secretion in the staurosporine-pretreated atrium (Figure 2(b)(D)).

### 3.3. Effect of Diltiazem on ELA-Induced Changes

**Figure 3:**
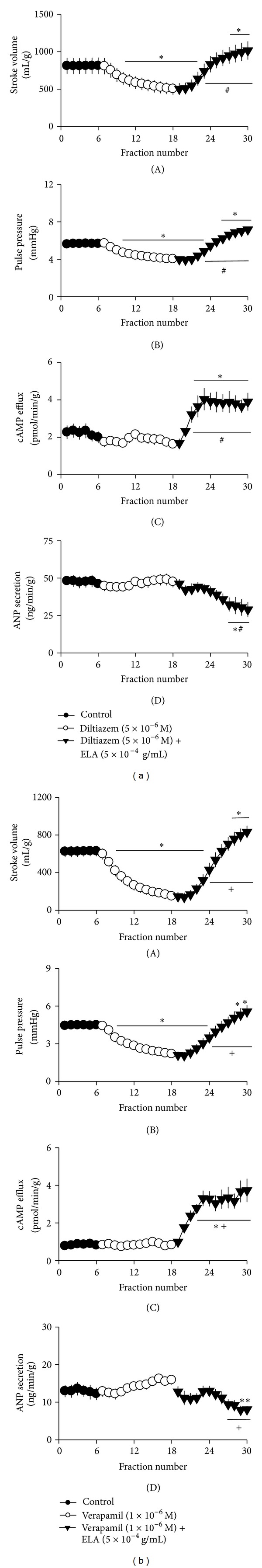
Effects of diltiazem (a) and verapamil (b) on ELA-induced changes in stroke volume (A), pulse pressure (B), cAMP efflux (C), and ANP secretion (D) in beating rabbit atria. Values shown are mean ± SE (*n* = 4); ***P < 0.001 versus control; ^###^ *P* < 0.001 versus diltiazem or verapamil (compared with values for the last 3 fractions of control, diltiazem, or verapamil).

To investigate whether Ca^2+^ channels are involved in the ELA-induced positive inotropic activity, diltiazem, an L-type Ca^2+^ channel blocker, was used to pretreat beating atria. Treatment of beating atrium with diltiazem (5 × 10^−6^ M) markedly decreased stroke volume and pulse pressure (Figures 3(a)(A) and 3(a)(B)). However, the diltiazem-induced reductions in atrial stroke volume and pulse pressure were reverted to levels greater than the basal levels by subsequent treatment with ELA (5 × 10^−4^ g/mL) (Figures 3(a)(A) and 3(a)(B)). As shown in Figure 3(a)(C), cAMP efflux level was not altered by treatment with diltiazem but increased by perfusion with ELA after the pretreatment with diltiazem (Figure 3(a)(C)). Diltiazem had no effect on ANP secretion in beating atria. However, ELA markedly decreased ANP secretion in the diltiazem-pretreated atrium (Figure 3(a)(D)).

### 3.4. Effect of Verapamil on ELA-Induced Changes

To confirm that L-type Ca^2+^ channels are involved in the ELA-induced positive inotropic effect, verapamil, another L-type Ca^2+^ channel blocker, was also tested. Treatment with verapamil (1 × 10^−6^ M) markedly decreased stroke volume and pulse pressure in beating rabbit atria (Figures 3(b)(A) and 3(b)(B)). However, subsequent treatment with ELA (5 × 10^−4^ g/mL) reverted the verapamil-induced decreases in atrial stroke volume and pulse to values higher than basal levels (Figures 3(b)(A) and 3(b)(B)). The cAMP efflux level was not altered by treatment with verapamil but was increased by perfusion with ELA after the verapamil treatment (Figure 3(b)(C)). ELA also decreased ANP secretion in the verapamil-pretreated atrium (Figure 3(b)(D)).

### 3.5. Change in the ELA-Induced K^+^ Concentration in Beating Atria-Derived Perfusate

**Figure 4:**
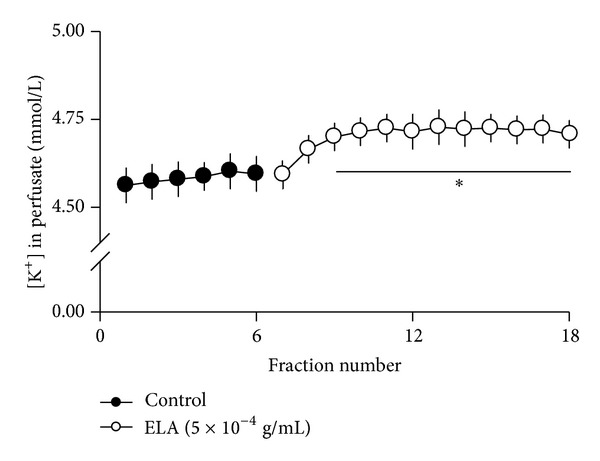
Change in ELA-induced K^+^ concentration in beating atria-derived perfusate. Values shown are mean ± SE (*n* = 4); ***P < 0.001, versus control (compared with values for the last 3 fractions of control).

The K^+^ concentration in the beating atria-derived perfusate was examined to evaluate whether Na^+^/K^+^-ATPase is involved in the ELA-induced positive inotropic activity. As shown in Figure 4, the K^+^ concentration in the beating atria-derived perfusate was markedly increased by perfusion with ELA. 

### 3.6. Effect of Helveticoside on Atrial Stroke Volume, Pulse Pressure, cAMP Efflux, and ANP Secretion

**Figure 5:**
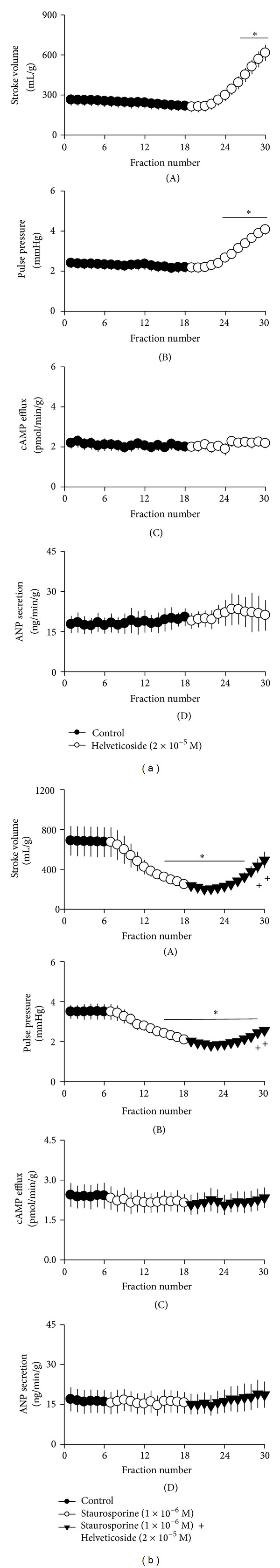
Effects of helveticoside (a) and staurosporine (b) on helveticoside-induced changes in stroke volume (A), pulse pressure (B), cAMP efflux (C), and ANP secretion (D) in beating rabbit atria. Values shown are mean ± SE (*n* = 4); **P < 0.01, ***P < 0.01 versus control; ^###^ *P* < 0.001 versus staurosporine (compared with values for the last 3 fractions of control or staurosporine).

To determine whether the ELA-induced positive inotropic effects are due to helveticoside, which is a digitalis-like cardiac glycoside constituent of *Lepidium apetalum*, the effects of helveticoside on beating rabbit atria were determined. Similar to the ELA-induced pattern, helveticoside (2 × 10^−5^ M) significantly increased stroke volume and pulse pressure (Figures 5(a)(A) and 5(a)(B)). However, there were no changes in cAMP efflux and ANP secretion after treatment with helveticoside (Figures 5(a)(C) and 5(a)(D)). The data were expressed Δ% changes of the mean values of fraction number 29/30 over the values of fraction number 17/18 (Figure 7).

### 3.7. Effect of Staurosporine on Helveticoside-Induced Changes

To define the roles of protein kinases in the helveticoside-induced positive inotropic activity, beating atria were treated with staurosporine. The atrial stroke volume and pulse pressure after treatment of beating atrium with staurosporine (1 × 10^−6^ M) were significantly lower than those in the controls (Figures 5(b)(A) and 5(b)(B)). When staurosporine-pretreated beating atria were treated with helveticoside (2 × 10^−5^ M), atrial stroke volume and pulse pressure reverted to levels higher than those observed after the staurosporine treatment alone (Figures 5(b)(A) and 5(b)(B)). Staurosporine had no effect on cAMP efflux and ANP secretion in beating atria. Treatment of helveticoside with staurosporine also caused no changes in cAMP efflux and ANP secretion in beating atria (Figures 5(b)(C) and 5(b)(D)). The data were expressed Δ% changes of the mean values of fraction number 29/30 over the values of fraction number 17/18 (Figure 7).

### 3.8. Effect of Ca^2+^ Channel Blockers on Helveticoside-Induced Changes

**Figure 6:**
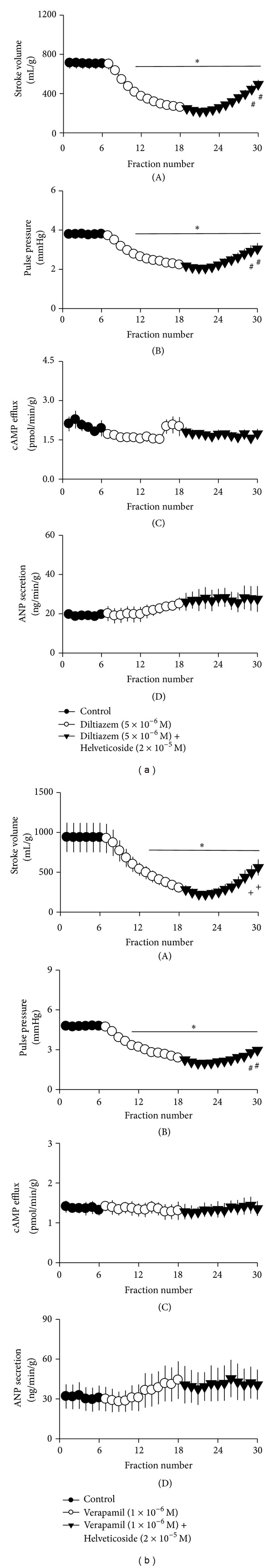
Effects of diltiazem (a) and verapamil (b) on helveticoside-induced changes in stroke volume (A), pulse pressure (B), cAMP efflux (C), and ANP secretion (D) in beating rabbit atria. Values are mean ± SE (*n* = 4); ***P < 0.001 versus control; ^###^ *P* < 0.001 versus diltiazem, or verapamil (compared with values of the last 3 fractions of control, diltiazem, or verapamil).

**Figure 7:**
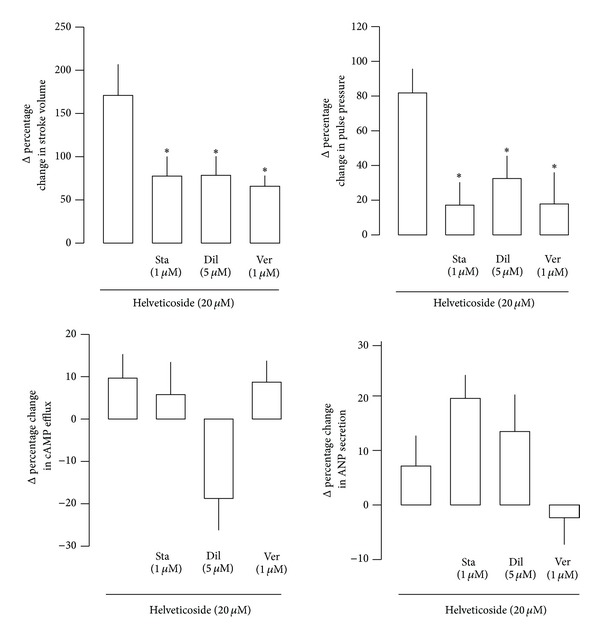
Effects of modulators on helveticoside-induced Δ% changes in pulse pressure, stroke volume, cAMP efflux, and ANP secretion. Data were derived from Figures 5 and 6. The values are Δ% changes of mean values of fraction number 29/30 over mean values of fraction number 17/18. Values are means ± SE. *P < 0.05 versus the mean values of two fractions before angles are changed (in modulators: staurosporine, diltiazem, and verapamil).

To investigate whether Ca^2+^ channels are involved in the helveticoside-induced positive inotropic activity, beating rabbit atria were pretreated with diltiazem or verapamil. Treatment of beating atrium with diltiazem (5 × 10^−6^ M) markedly decreased stroke volume and pulse pressure (Figures 6(a)(A) and 6(a)(B)). However, the diltiazem-induced reductions in atrial stroke volume and pulse pressure were significantly reverted by subsequent perfusion with helveticoside (Figures 6(a)(A) and 6(a)(B)). Verapamil (1 × 10^−6^ M) also markedly decreased stroke volume and pulse pressure (Figures 6(b)(A) and 6(b)(B)), which recovered upon subsequent treatment with helveticoside (2 × 10^−5^ M) (Figures 6(b)(A) and 6(b)(B)). Diltiazem and verapamil had no effect on cAMP efflux and ANP secretion in beating atria. Treatment of helveticoside with diltiazem or verapamil also caused no changes in cAMP efflux and ANP secretion in beating atria (Figures 6(a)(C) and 6(a)(D), 6(b)(C), and 6(b)(D)). The data were expressed Δ% changes of the mean values of fraction number 29/30 over the values of fraction number 17/18 (Figure 7).

## 4. Discussion

This study clearly shows that ELA increases stroke volume, pulse pressure, and cAMP efflux in beating rabbit atria. Because the cAMP-signaling pathway modulates the activation of L-type Ca^2+^ channels and PKs, leading to accentuation of cardiac contractility in beating atria [9, 16–18], it was expected that cAMP would be involved in the ELA-induced increase in atrial dynamics via the L-type Ca^2+^ channels and/or PKs. However, our results showed that blocking of L-type Ca^2+^ channels with diltiazem or verapamil had no effect on the ELA-induced increases in stroke volume, pulse pressure, and cAMP efflux in beating rabbit atria. Similarly, inhibition of PKs with staurosporine did not affect the ELA-induced increases in stroke volume, pulse pressure, and cAMP efflux. It has been reported that *Convallaria keiskei*, which contains the cardiac glycoside-like molecule convallatoxin, increases stroke volume and pulse pressure without an associated increase in cAMP efflux in the perfusate [19]. These results suggest that ELA-induced positive inotropic activity is not caused by the pathway mediated by L-type Ca^2+^ channels and/or protein kinases.

Stimulation of *β*-adrenergic receptors with a sympathomimetic agent induces positive inotropic effects that are dependent on PKs and L-type Ca^2+^ channels [2, 3]. Stimulation of *β*-adrenergic receptors results primarily in an increase in cAMP production and consequent activation of PKs and phosphorylation of L-type Ca^2+^ channels, thereby further increasing the channel open time and/or the probability of opening of functional Ca^2+^ channels [20]. Increase in the cAMP level by PDE III inhibitors also accentuates cardiac contractility via activation of PKs and L-type Ca^2+^ channels [4]. In accordance with our hypothesis, the ELA-induced positive inotropic effect was not altered by pretreatment with L-type Ca^2+^ channel blockers and a protein kinase inhibitor. These findings suggest that the activities of sympathomimetics and PDE III inhibitors could be excluded from the possible mechanism of the ELA-induced positive inotropic effect.

We also determined the effects of helveticoside on atrial dynamics, cAMP efflux, and ANP secretion in beating rabbit atria. Helveticoside, the main constituent of ELA, is a well-known digitalis-like compound. Similar to digitalis, helveticoside markedly increased the pulse pressure and stroke volume, without increasing cAMP efflux, in beating rabbit atria. Helveticoside also increased pulse pressure and stroke volume in the staurosporine-pretreated atria. Furthermore, helveticoside induced positive inotropic activity in diltiazem- and verapamil-pretreated atria. In cAMP and ANP regulation, we cannot rule out other component's possibility from ELA except for helveticoside. Thus, further study is needed to clarify the effect of linoleic acid or olein on the cAMP efflux and ANP secretion.

A previous report suggested that helveticoside could inhibit Na^+^/K^+^-ATPase activity in an *in vitro* enzyme assay [21]. In this study, we show that treatment with ELA markedly increased K^+^ concentration in beating atria-derived perfusate. Many lines of evidence have demonstrated that digitalis-like cardiac glycosides increase cardiac contractility by elevating intracellular Ca^2+^ concentration via Na^+^/K^+^-ATPase inhibition-mediated activation of Na^+^/Ca^2+^ exchanger [6, 22, 23]. In this case, the K^+^ efflux would be increased because K^+^ influx is inhibited due to the inhibition of Na^+^/K^+^-ATPase in the myocardium. In our study, ouabain markedly increased the pulse pressure and stroke volume without increasing the cAMP efflux in beating rabbit atria, resulting in a positive inotropic effect via inhibition of Na^+^/K^+^-ATPase activity. These findings suggest that the digitalis-like activity of helveticoside might be associated, at least in part, with ELA-induced positive inotropic activity.

The heart is also an endocrine gland, secreting ANP, which is involved in the regulation of body fluid and blood pressure [11, 24]. The present study shows that ELA significantly decreased ANP secretion in beating rabbit atria. Diltiazem or verapamil, but not staurosporine, slightly increased ANP secretion in beating rabbit atria. The ELA-induced reduction in ANP secretion was not affected by pretreatment with diltiazem, verapamil, or staurosporine. The potential roles of cyclic nucleotide and Ca^2+^ in the regulation of ANP release have been the subject of interest of many studies. cGMP and Ca^2+^ inhibit ANP secretion in perfused atria [25]. On the other hand, cAMP increases the ANP secretion in rat cardiomyocytes [26], isolated atrium [27], and perfused rat atria [28]. However, there are diverse reports on the effects of cAMP in the regulation of ANP secretion. Forskolin, an adenylyl cyclase activator, has been shown to decrease ANP secretion from cultured atrial myocytes [29, 30] and in perfuse beating rat hearts [31]. Likewise, 3-isobutyl-1-methylxanthine (IBMX) and 8-bromoadenosine 3′,5′-cyclic monophosphate (8-BrcAMP), a nonselective PDE inhibitor and a cAMP agonist, respectively, inhibit ANP secretion [30, 31]. Collectively, ELA significantly decreased the ANP secretion associated with increase in cAMP efflux in beating rabbit atria, consistent with other reports [26, 30, 31].

Taken together, the present study suggests that the ELA-induced positive inotropic activity may, at least in part, be due to inhibition of Na^+^/K^+^-ATPase activity by helveticoside-like cardiac glycosides.
